# Intraoperative techniques in realtime ureteric navigation. A brief narrative review and a video vignette

**DOI:** 10.3389/fsurg.2025.1647010

**Published:** 2025-09-25

**Authors:** Jayan Dewantha Jayasinghe, Valentin Butnari, Kylin Weiguang Hu, Mohamed Adhnan Thaha

**Affiliations:** 1 Department of Colorectal Surgery, The Royal London Hospital, Barts Health NHS Trust, London, United Kingdom; 2 The Centre for Neuroscience, Surgery and Trauma, Blizard Institute, Faculty of Medicine and Dentistry, Queen Mary University of London, National Bowel Research Centre, London, United Kingdom

**Keywords:** navigation, ureter identification, fluorescence-guided surgery, indocyanine green, colorectal surgery, laparoscopy, robotic surgery

## Abstract

**Background:**

Intraoperative ureteric injury, a well-documented but avoidable adverse event in pelvic surgery, is sometimes associated with low surgical volume and inexperience of the surgeon. The current literature describes several techniques that can help surgeons identify and protect the ureter during pelvic dissection, especially during complex procedures or repeat surgeries. This narrative review aims to highlight and present the currently available techniques for intraoperative ureteric identification in colorectal surgery and showcase the use of Indocyanine Green (ICG) in real-time ureter identification using a video vignette.

**Methods:**

A literature search of the PubMed database was performed from inception until May 14, 2025, to identify relevant articles reporting on intraoperative ureteric navigation techniques in colorectal surgery. Articles were analysed if they described the application of a technique in colorectal surgical procedures to identify the ureter intraoperatively. Given the narrative nature of this review, a qualitative synthesis was conducted by identifying the key themes described. In a video, we demonstrate a case of laparoscopic Hartmann's reversal of how ICG can facilitate surgical steps and the outcomes of a challenging procedure.

**Results:**

Techniques to identify the ureter intraoperatively during colorectal surgery were identified and discussed: (1) Image-Guided Techniques comprising Fluorescence Imaging and Use of Intraoperative Ultrasound; (2) Computer-Assisted and Augmented Reality Navigation consisting of 3D Model-Based Navigation, Augmented Reality, and Artificial Intelligence; and (3) Mechanical or Physical Identification Aid. These techniques highlight the improved outcomes in complex colorectal surgeries by preventing ureteric injury while enhancing the training process, even in complex, redo surgeries.

**Conclusion:**

Our narrative review highlights that image-guided surgery and augmented reality techniques are rapidly evolving in colorectal surgery. Our video vignette shows that intraoperative ureteric navigation makes challenging adhesiolysis easier and safer, which subsequently facilitates the training process in benign complex or re-do minimally invasive pelvic surgery.

## 1 Introduction

Advanced minimally invasive pelvic surgeries, including colorectal, gynaecological, and urological procedures, require exceptional technical proficiency and seamless coordination of the whole team. Accurate identification of the correct anatomical planes requires higher levels of anticipatory skills in the context of a hostile abdomen and pelvis and an altered anatomy in these reoperative cases. Amongst the key concerns during such challenging surgeries is the need to avoid inadvertent iatrogenic injuries to nearby vital structures.

Iatrogenic ureteric injury during laparoscopic colorectal surgery is reported in the range of 0.2%–1.0%, and although this is rare, approximately 80% of them occur intraoperatively (1–4). This relatively uncommon but significant complication has considerable morbidity, mortality, and impact on the quality of life (5–7) of patients, and some studies suggest that its incidence may be increasing (2, 8). In colorectal surgery, ureteric injuries are more common during left colonic surgery for benign indications, such as diverticulitis and its complications, Crohn's disease, and following preoperative radiotherapy or previous abdominopelvic surgery (3–7). When it occurs, the vast majority of these injuries (50%–70%) are only recognized postoperatively, with a reported median delay of 10 days from the index procedure (7–10). Iatrogenic ureteric injuries have also been reported during laparoscopic surgery for non-gastrointestinal procedures, such as gynaecological surgery, transplant and urological surgery, and pelvic lymph node dissection for malignancy (3, 8, 11). Although various predisposing factors for iatrogenic injury have been reported in the literature, currently there is not a single non-invasive prevention strategy that effectively reduces the risk (12).

This manuscript presents a brief narrative literature review describing the currently available techniques for intraoperative ureteric identification, together with an illustrative video vignette of a complex, laparoscopic re-do pelvic case carried out by a senior colorectal trainee where intraoperative, dynamic ureteric navigation was achieved using fluorescence-guided NIR (Near Infrared) surgery imaging after intra-ureteric instillation of Indocyanine green (ICG).

## 2 Material and methods

### 2.1 Narrative review

A literature search was performed to identify relevant articles on the application of intraoperative ureteric navigation techniques. The PubMed database was searched from its inception until [May 14, 2025]. Medical Subject Headings (MeSH) terms were used in various combinations with the application of Boolean operators (AND, OR) to refine the search strategy. The key MeSH terms used included “ureter”, “intraoperative”, “visualization”, “navigation”, “imaging”, and “injury.” Articles that focused on the application of navigation techniques for ureteric identification in colorectal surgical procedures were reviewed. Given the narrative nature of this review, a qualitative synthesis was performed by identifying the key themes presented in the discussion (13).

### 2.2 Video vignette

Our patient, a 58-year-old Caucasian male with a Performance Status of 0 and a body mass index of 31 kg/m^2^, had previously undergone emergency Hartmann's procedure for Hinchey class III diverticular disease. A follow-up completion colonoscopy through the stoma did not reveal any other bowel abnormality, and flexible endoscopy per anus revealed an unremarkable 20 cm long rectal stump. His anaesthetic fitness was confirmed, in preparation for an elective procedure to restore intestinal continuity. A senior colorectal trainee performed laparoscopic reversal of Hartmann's procedure with remote consultant supervision (Supplementary Video S1). Informed consent was obtained for video recording and publication before surgery.

## 3 Results

### 3.1 Narrative review

**Figure 1:**
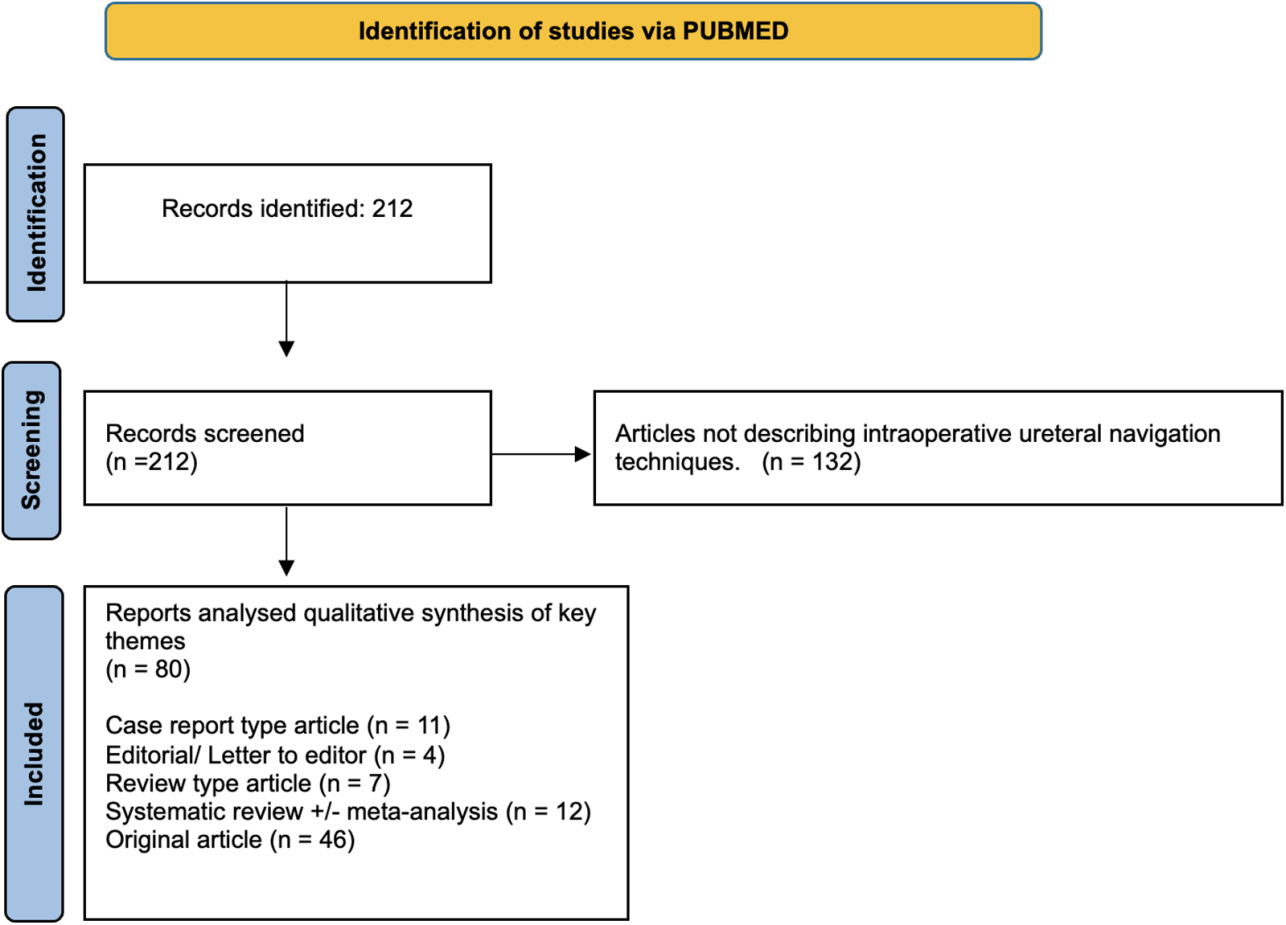
PRISMA Flow Diagram.

The systematic PubMed search identified 212 studies of which 80 described various techniques for intraoperative ureteric identification. These included: 11 case reports, 4 editorials or letters to the editor, 7 review articles, 12 systematic reviews (with or without meta-analysis), and 46 original articles (Figure 1). Full texts were analysed to identify common themes. We identified three main themes: Mechanical/Physical Identification Aids; Image-Guided Techniques; and Computer-Assisted & Augmented Reality (AR) navigation.

Image-Guided Techniques described within the articles from our search are predominantly focused on advanced visualization methods with Fluorescence Imaging using agents such as ICG and Methylene Blue, and a smaller number of studies reported on the use of emerging novel dyes. Minimal data is currently reported on Intraoperative use of Ultrasound to identify the ureter. Second main theme is represented by Computer-Assisted & Augmented Reality (AR) Navigation (emerging in MIS), this includes 3D model-based systems, AR, and the growing impact of AI/ML for image recognition. Last but not least, Mechanical/Physical Identification Aids like lighted and conventional ureteral stents are well established methods which are widely available around the world.

### 3.2 Surgical technique

The surgical technique used in the case is described in the video vignette (Supplementary Video S1). This is a practical demonstration of how ureteric navigation techniques can be effectively used in the context of a reoperative complex colorectal case performed for benign indications. Preoperative renal and liver function tests were unremarkable, and contrast allergies were excluded, making the surgical case suitable for ICG administration.

After induction of anaesthesia, a rigid cystoscope was used in the operating room, followed by fluoroscopy-guided retrograde advancement of a 6 Fr open-ended ureteral catheter (Cook Medical, Bloomington, IN, USA) over a guidewire, with the catheter tip positioned in the left renal pelvis. ICG was prepared by dissolving 25 mg ICG in a single vial in 10 ml sterile water. 5 ml of the ICG solution was injected through the ureteric catheter, via the distal end external to the urethral orifice. The free end of the ureteric catheter was introduced into the urethral Foley catheter and clamped during surgery.

The patient was then placed in the Lloyd-Davies position with the surgeon operating from the right side of the patient, and the first assistant standing at the right shoulder behind the surgeon and the laparoscopic monitor at the level of the left pelvis, following the standard setup for a left-sided colorectal surgery. The colostomy site was prepared with antiseptic solution and the whole abdomen was covered with antimicrobial incise drape, which provides a barrier against microbial contamination. An open cut-down technique at the umbilicus was used to safely access the peritoneal cavity and create pneumoperitoneum. A standard three-port setup was used for adhesiolysis with two 5 mm ports inserted under direct vision in the right iliac fossa and right mesogastrum. Near-infrared (NIR) visualization was performed using an Arthrex® Synergy ID™ imaging system (SynergyID system by Arthrex, Naples, FL, USA) with a 10 mm 30^0^ camera. The left ureter was clearly visualized from the outset and throughout the procedure, tracing its course along the left posterolateral abdominal wall, over the pelvic brim, and into the left pelvic side wall. This was particularly helpful during adhesiolysis as well as mobilization of the small bowel, colon, and rectal stump in the context of previously advanced inflammatory reactions. Following a complete adhesiolysis and full mobilization of the left colon, the colostomy was detached, and the 29 mm anvil of a circular stapler was secured at the colostomy opening and dropped inside the peritoneal cavity. The colostomy wound was protected with a small Alexis^TM^ wound protector, and the pneumoperitoneum was re-established. An end-to-end, tension-free stapled colorectal anastomosis was performed to restore intestinal continuity. Anastomotic integrity and health was confirmed with a negative leak test, two complete doughnuts and intravenous indocyanine green (ICG) demonstrated satisfactory perfusion.

At the end of surgery, the ureteric catheter was removed without further urinary tract intubation.

The total operative time was 90 min, and the estimated blood loss was less than 100 ml without perioperative transfusion requirements. The patient had an uneventful postoperative course with no postprocedural urinary symptoms, infections, or anastomotic failure. He successfully passed the trial without catheter on post operative day one and was discharged on the 4th postoperative day. At the 3-month follow-up, no postoperative complications were recorded.

## 4 Discussion

The increased complexity and widespread adoption of laparoscopic abdominal and pelvic surgery have unfortunately coincided with a rise in the iatrogenic ureteric injury rate (8). Our literature review systematically explored the developed preventive methods and intraoperative strategies developed to mitigate these injuries. The increasing use of laparoscopy and other minimally invasive approaches have popularized these techniques.

### 4.1 Mechanical/physical identification

Mechanical/Physical Identification Aids, such as conventional and lighted ureteral stents, are well-established methods available worldwide. Prophylactic preoperative ureteric stenting (PUS) involving single or bilateral ureters has long been a common practice in complex open surgery, facilitating positive identification through palpation and serving as a useful aid in the event of iatrogenic injury requiring repair (14, 15). More recent studies with large patient cohorts also suggest similar benefits of PUS in laparoscopic colorectal surgery too (16–18). However, its effectiveness in minimally invasive surgery has been questioned by others because of the lack of haptic feedback (19–21). Selective PUS is often reported in patients undergoing left sided resections for complicated diverticular disease or obesity where identification of ureter is not without drawbacks (15, 19–21). PUS can lead to urological morbidities, such as urinary tract infections, haematuria, reflux anuria, hydronephrosis, perforation, and prolonged operating time (17–19). Furthermore, patients often require a second cystoscopy for postoperative stent retrieval, in addition to costs, waiting times, and infection risks.

The first reported use of lighted ureteric stents and catheters (LUS) to aid ureteric identification in both open and laparoscopic surgery was in 1994 (4, 22–24), and studies have shown promising results in preventing ureteric injury with LUS in laparoscopic colorectal surgery (25–28). Nevertheless, LUS has disadvantages similar to those of conventional ureteric stents, including morbidities associated with ureteric instrumentation, increased operating time, additional costs, and inconvenience of a second cystoscopy for stent retrieval (4, 23). Currently, no randomized trials have compared conventional preoperative ureteric stents with LUS for visualizing, preventing, and early detection of iatrogenic ureteric injury during laparoscopic benign colorectal surgery (23, 28).

### 4.2 Image-guided techniques

This group of techniques, particularly dominant in MIS, primarily focuses on advanced visualization. The most commonly described fluorophores in use for Fluorescence Imaging are ICG and Methylene Blue. Dye-based techniques have helped to overcome the lack of haptic feedback and the inability to directly palpate the ureteric stents during laparoscopic surgery, and are increasing in popularity.

Indigo Carmine, also known as indigotindisulfonate sodium, is one of the oldest drugs used as a contrast agent for ureteric navigation. This drug, used for over eight decades, is a blue dye injected intravenously into a peripheral vein during surgery, which is then excreted in the urine, and the stained urine can be detected using standard laparoscopic vision (22). The technique is most commonly used to evaluate the patency of the urinary tract and to identify urine leaks following injury to the ureters and/or bladder, but has become mostly redundant due to multiple issues, including anaphylactic reactions (22, 29–31).

#### 4.2.1 Methylene blue

Methylene Blue (MB) is an intravenous fluorophore injected peripherally and visualized using NIR fluorescence-enabled systems. As demonstrated in clinical studies, MB can be administered at doses of 0.25 mg/kg–1 mg/kg. In open surgery, the optimum dose was stated as 0.25 mg/kg with no statistically significant improvement in Signal-to-background ratio (SBR) when higher doses were given (32). The optimum dose for injection in laparoscopic surgery is 0.75 mg/kg which delivers a high SBR with slower SBR decline over time (32–34). Once administered, fluorescence was observed in the ureters within 10 min, and remained detectable for up to 60 min. In one study, the longest fluorescence was observed at 2 h and 5 min. Studies have shown an inverse relationship between dosage and SBR decline over time, whereby a higher dose (e.g., 1 mg/kg) is associated with a slower rate of SBR decline (33).

There are many advantages to using MB as a contrast agent. With a fluorophore emission at an estimated 700 nm, MB demonstrates a relatively high tissue penetrance of 3–5 mm, allowing its use in a wide range of procedures (32). MB exhibits both renal and hepatic clearance. Hence, it is suitable for IV administration; this makes the setup for ureter visualization significantly more practical and time efficient when compared to ureteric stenting. Another advantage of MB is that it enables ureter visualization, even at low doses, thereby mitigating any potential adverse effects. No adverse outcomes were reported in any of these studies (32).

There are some limitations to MB. First, the excitation and emission of MB (688 nm) fall within the visible light spectrum (400–700 nm), potentially impacting the displayed image due to autofluorescence (32). The contrast is cleared through the renal system and hence, cannot be given to patients with poor renal function. Further, as urine flow is non-continuous, it gives a pulsatile, rather than continuous, visualization of the renal system. The use of diuretics to improve urine flow did not result in an increase in the NIR fluorescence. Given that the ureter is a retroperitoneal structure, a tissue penetrance of 3–5 mm may be insufficient in some cases. Pregnancy, previous hypersensitivity or anaphylactoid reactions, and glucose-6-phosphate dehydrogenase deficiency are generally considered contraindications for MB use. Patients with glucose-6-phosphate dehydrogenase deficiency are susceptible to developing haemolytic anemia (35). Finally, MB is known to contribute to serotonin syndrome, so it should be avoided in patients taking SSRIs, SNRIs, TCAs, and MAOIs. Although MB can be toxic at high doses, toxicity does not occur at doses <2 mg/kg (35).

#### 4.2.2 Indocyanine green (ICG)

In our literature review, ICG emerged as the preferred fluorophore for ureter identification. Unlike other applications where ICG is typically administered intravenously, the method for ureter identification involves direct instillation into the ureter as it is excreted through bile, not the urinary tract, after intravenous injection. Siddighi et al. (36) describes a technique similar to what we used in the case reported here. For visualizing the entire ureter, the catheter tip is positioned at the renal pelvis for anterograde ICG instillation. However, if only the pelvic ureter needs visualization, a more limited catheterization, with the tip at the ureteral orifice, suffices. The ureteral fluorescence persists throughout the operation as clamping the ureteric catheter limits fluorophore drainage and is less dependent on renal flow. In all studies, ICG was considered safe and effective for identifying the ureters and preventing ureteral injury.

The main limitation of ICG for ureteral visualization is its hepatic clearance. A ureteral catheter is required, which carries the risk of iatrogenic injury and increases surgical setup time. This set up time further varies depending on the extent of catheterization required for adequate visualization (36). Although generally well tolerated, ICG use requires caution in patients with liver disease. Pregnancy is a relative contraindication for ICG administration. It should be fully avoided in patients with uraemia, previous hypersensitivity reactions, and known allergies to iodide.

ICG demonstrates an excellent safety profile, characterized by a low incidence of adverse reactions, making it highly suitable for various surgical applications (37–40). This is further supported by its longer fluorescent duration, rapid hepatic clearance, and the ability to administer additional doses without interference. Commonly used at 0.1–0.5 mg/kg for non-ureteric visualization, ICG operates safely within established dose limits (41). ICG rapidly binds to plasma proteins, undergoes hepatic clearance, and appears in bile within 10–15 min without metabolism. Its utility extends across both laparoscopic and open surgical settings (39).

#### 4.2.3 Novel fluorescent substances

Methylene Blue and ICG are the only commercially available NIR fluorescent agents approved by the Food and Drug Administration and European Medicines Agency (22). Each has distinct advantages and disadvantages for ureter visualisation. Several promising novel dyes for ureter visualization are in development, aiming for prolonged fluorescence, rapid renal excretion, excitability by standard light sources, and an improved safety profile (42).

#### 4.2.4 ZW800-1

ZW800-1 is a promising fluorescent dye that has demonstrated precise delineation of ureters in human clinical studies. It is a small zwitterionic molecule exhibiting a neutral electrical charge, resulting in extremely low non-specific binding and tissue uptake. This is a fundamental problem with conventional dyes, which are highly anionic, improving solubility but resulting in high non-specific tissue uptake (43). ZW800-1 has good tissue penetrance and allows ureter identification without further tissue dissection, even at low doses (1.0–2.5 mg). At a very low dose (2.5 mg), ZW800-1 allowed imaging for more than 3 h and reached a peak signal intensity within 10 min. With near-exclusive renal clearance, it gives off a high Signal Background Ratio (SBR) peaking at 75.0, owing to its low background fluorescence. This fluorophore was engineered to allow one-step conjugation with peptides such as cRGD-ZW800-1. Human studies have demonstrated complete clinical translation, providing proof of its safety and efficacy. It can be used with multiple surgical fluorescence imaging systems, including Olympus, Da Vinci, Firefly and FLARE (22, 44).

#### 4.2.5 IRDye CW800BK

Another dye currently undergoing clinical translation is the IRDye CW800BK. Following several porcine studies, this dye demonstrated clear delineation of the ureters within 5 min of intravenous administration, and remained visible for at least 60 min (45). With an emission wavelength of 790 nm, there was less visual disturbance from autofluorescence, and the predominant renal clearance prevented background fluorescence. This contributed to the higher SBR (22). CW800BK was directly compared to several competing fluorescent dyes. In a cadaver study visualizing the urethra, CW800BK was considered a promising alternative to ICG while proving superior in both SBR and depth of tissue penetration at 2 cm (43). When compared with other novel agents, IRDye 800CW and IRDye 800NOS, IRDye CW800BK had the highest measured target-to-background ratio (TBR) (46). The unique advantage of CW800BK is that it has both biliary and renal clearances. Al-Taher demonstrated that this allows simultaneous assessment of the ureter, bile ducts, bowel perfusion, and lymphography, making this fluorophore a versatile surgical tool (47). Another fluorophore, CW800-CA, has been used in clinical trials. It is a carboxylate version of IRDye800CW NHS-Ester that allows renal clearance and has been shown to a higher SBR than CW800BK (22).

#### 4.2.6 Other novel fluorescent agents

Several novel fluorophores have been used in preclinical studies, and we will explore them briefly. UreterGlow -11 is a PEGylated version of UreterGlow that improves prolonged circulation and reduces non-specific tissue uptake. It is cleared through the renal system and has been demonstrated in murine studies to provide strong ureteral fluorescence for at least 12 h. The emission wavelength of the dye was intentionally shifted by∼30 nm to allow differentiation of the ureters against other major imaging dyes. Although deemed a good candidate, there are currently no clinical trials (42). Another novel fluorescent agent is UI-766. This dye showed exclusive renal clearance, outperforming CW800 with an SBR that was two times higher. This dye has been studied in porcine models, achieving ureter visualization within 10 min of injection and delivering a fluorescent signal for at least 4 h. Currently, no clinical trials have been conducted (44, 48). Liposomal ICG is a formulation of ICG encapsulated in liposomes that enhances renal clearance. However, because it is still partially excreted by the liver, it can result in a high background fluorescence, limiting its imaging specificity. In murine studies, ureters can be visualized 10 min after injection and can last for 90 min. Liposomal formulations may have genotoxic effects, as observed in rat studies. No clinical trials are underway (22). Fluorescein and Genhance 750 are novel agents studied in animals. Although fluorescein can be used safely to visualize ureters in animals, the studies do not mention SBR or the duration of fluorescence. Since fluorescent emission occurs at 520 nm, which is within the visible light spectrum, we can expect low tissue penetration and high autofluorescence. Little is known about the benefits of Genhance 750 and its comparison with other dyes (22).

### 4.3 Computer-assisted and augmented reality (AR) navigation

This group includes emerging Minimally Invasive Surgery (MIS) techniques such as 3D model-based systems, augmented reality (AR), and Artificial Intelligence/Machine Learning (AI/ML) for image recognition. Virtual Reality (VR)-enhanced imaging has shown promise in improving patient communication and consent in colorectal surgery, and in reducing preoperative anxiety through immersive simulation (49–51). Positioned on the “virtuality continuum,” AR overlays 3D anatomical data onto the real world, and viewed via devices like the HoloLens 2 (52, 53). However, a key limitation remains: preoperative radiological images are static, while surgical anatomy is dynamic and deformable. This mismatch can reduce the accuracy of VR-assisted navigation, particularly in tasks like ureteric identification. Combining VR with AI offers a promising solution—algorithms like *UreterNet*, trained on over 14,000 annotated frames from 304 colorectal videos, can accurately identify the ureter in real time (71 ms inference and 143 ms for output), enhancing intraoperative safety and precision (54). In parallel, the increased availability of near-infrared (NIR) fluorescence imaging in MIS platforms enables real-time visualisation of ureters (55, 56). Although early concerns about thermal injury from NIR exposure have been reported in animal studies, modern laparoscopic systems mitigate this risk (57). Collectively, these technologies support precise ureteric identification, improve training, and contribute to safer surgical outcomes.

### 4.4 Limitations and future directions

Our literature review highlighted a diverse range of techniques for intraoperative ureteric navigation. There are no published randomized control trials comparing preoperative ureteric stenting and fluorescence-guided techniques for visualizing the ureter in open or laparoscopic surgery. No direct comparisons exist between different methods of fluorescence ureterography, including ureteric ICG, intravenous methylene blue, and lighted ureteric stents (LUS). The impact of these techniques on the overall procedure time or surgeons' learning curve are under-reported. The use of intraoperative ICG is increasing in popularity as more NIRS equipped MIS platforms become readily available, and could make a positive impact on surgeon training in complex MIS without compromising patient safety. The additional value of combining ICG and prophylactic ureteric stenting is unproven. Despite these developments, intraoperative ureteric injury remains a significant complication, which should be avoided. Our narrative review underscores the critical role of advanced visualization techniques in mitigating this risk, particularly in complex, re-do surgery. Integrating augmented reality and AI offers significant potential for advancing ureteric navigation in abdominal and pelvic surgery. These technologies enable more intuitive, accurate guidance, enhancing safety in complex MIS. As shown in our video vignette, real-time ureteric identification with ICG aids challenging adhesiolysis in benign surgery, improving outcomes and serving as a valuable training tool for pelvic procedures.

## 5 Conclusion

Ensuring patient safety by minimising iatrogenic injury must remain a surgical priority. This involves following standardised techniques, preparing contingency plans, viewing early stent placement as a proactive step, and utilising fluorescence for enhanced visualisation. Emerging technologies like augmented reality and artificial intelligence offer a transformative solution to the longstanding challenge of intra-operative ureteric identification, improving precision, efficiency, and safety in colorectal surgery.
